# Healthcare Provider Knowledge, Attitudes, and Practices in Hospice Care and Their Influencing Factors: A Cross-sectional Study in Shanghai

**DOI:** 10.34172/ijhpm.2022.6525

**Published:** 2022-08-15

**Authors:** Xiaohan Teng, Maocheng Tang, Limei Jing, Yifan Xu, Zhiqun Shu

**Affiliations:** ^1^School of Public Health, Shanghai University of Traditional Chinese Medicine, Shanghai, China.; ^2^Longhua Hospital, Shanghai University of Traditional Chinese Medicine, Shanghai, China.; ^3^Department of Traditional Chinese Medicine, Shandong College of Traditional Chinese Medicine, Yantai, China.; ^4^Shanghai Ninth People’s Hospital, Shanghai Jiaotong University School of Medicine, Shanghai, China.

**Keywords:** Hospice Care, Knowledge, Attitudes, Confidence, Practices, Shanghai

## Abstract

**Background:** In 2017, the Chinese government launched a pilot project in hospice care (HC), in which Shanghai was a pioneer. Healthcare provider knowledge, attitudes, and practices in hospice care (KAPHC) may facilitate or hinder HC development. To determine how to design targeted training for healthcare providers and policies to improve their KAPHC, we conducted an original study based on an indigenized KAPHC scale to (*a*) comprehensively measure the KAPHC baseline of healthcare providers in Shanghai and (*b*) explore its influencing factors.

**Methods:** A cross-sectional study was designed to evaluate healthcare providers’ KAPHC with the indigenized KAPHC scale. Descriptive analysis, linear regression, and Pearson’s (*r*) correlation analysis were performed to uncover providers’ KAPHC, its influencing factors, and their correlations.

**Results:** The KAPHC scale proved applicable to the knowledge, attitudes, and practices of the large sample of providers we surveyed. The 7027 KAPHC scaling results revealed that 42.44% of participants had HC experience and 57.49% were willing to provide HC. The mean accuracy of the responses related to knowledge was 59.30%. Scoring rates for attitudes, confidence, and self-reported practices were 74.20%, 73.96%, and 75.55%, respectively. The linear regression revealed that higher KAPHC scores were associated with experience and willingness and varied with professional specializations. The Pearson’s (*r*) correlation evidenced that HC practices were strongly correlated with confidence (*r* = 0.648, *P*<.001) and moderately correlated with attitudes (*r* = 0.463, *P*<.001).

**Conclusion:** We uncovered that targeted training for enhancing healthcare provider KAPHC in Shanghai should focus on psychological and spiritual care, the management of pain and other symptoms, and traditional Chinese medicine (TCM). Additionally, training should be scaled up for providers with different characteristics. Meanwhile, policy should encourage providers to work enthusiastically in HC—universal high-quality HC requires well-trained, supported, and motivated providers.

## Background

**Key Messages**

** Implications for policy makers**

- This was an original large-scale empirical study that evaluated healthcare provider knowledge, attitudes, and practices in hospice care (KAPHC) in China, based on an indigenized KAPHC scale published by our research team.
- In particular, this study focused on the knowledge, attitudes, and practices (KAP) of healthcare providers as a prerequisite to improve hospice care (HC) quality and provides an evidence-based scale and data useful for measurement in other areas of China.
- Health providers’ self-reported practices in HC were strongly correlated with confidence and moderately correlated with attitudes.
- There is an urgent need to enhance targeted training for healthcare providers across different skills.
- Policy-makers can formulate policies based to the results of this study, which may include targeted training for healthcare providers to ensure high-quality HC for patients with advanced and incurable diseases.

** Implications for the public**

Hospice care (HC) is a crucial part of integrated, people-oriented healthcare services. Providing physical, mental, spiritual, and social care for older people and terminally ill patients by treating their discomfort are the basic skills of healthcare providers working in this context. By completing training in the knowledge, attitudes, and practices (KAP) related to HC, healthcare providers can better satisfy their patients’ complex needs and improve their quality of life. Additionally, healthcare providers able to effectively apply their medical specializations in delivering integrated and people-oriented healthcare can benefit the public by enhancing the quality of life of terminal patients and helping them die peacefully with dignity. Moreover, improving healthcare providers’ knowledge, attitudes, and practices in hospice care (KAPHC) will enhance relationships between healthcare providers and patients.

Aging populations have become a tough challenge for many countries; they can yield complex and diversified demands for healthcare,^1^ including end-of-life (EOL) hospice care (HC) and palliative care. China has the largest elderly population in the world.^2^ According to the seventh census of China, people aged 60 and over comprised 18.70% of the total population in 2020.^3^ Meanwhile, Shanghai is home to an even higher percentage of older adults, with people aged 60 and over comprising 23.38% of the city’s population.^4^ China’s large aging population has given rise to an increasing demand for high-quality HC. The Economist Intelligence Unit’s2015 Quality of Death Index ranked mainland China 71 out of 80 countries^5^; this indicates that HC in China is limited in terms of availability and quality. In addition, due to the miniaturization of family structure and traditional Chinese lifestyles, most EOL patients do not receive sufficient treatment for pain and other physical, psychological and spiritual issues.^6^ The rapid global spread of coronavirus disease 2019 (COVID-19) and the great suffering it caused have emphasized the need to improve the functionality of HC—in May 2020, the World Health Organization (WHO) added palliative care to its Clinical Management of COVID-19 Interim Guidance.^7^ Notably, HC can not only significantly reduce the costs of caring for terminal patients but also produce considerable economic benefits.^8^

 HC and palliative care are crucial parts of comprehensive, people-oriented health services; more specifically, they integrate physical, mental, spiritual, and social care for terminal patients by controlling their discomfort.^9^ “HC” refers to the active holistic care given to individuals suffering from severe illness and EOL patients to improve their quality of life as well as that of their families.^10^ HC involves treating pain and other symptoms to ensure the patient is as comfortable as possible. Typically, patients and their families are provided psychological and social support in step with a multidisciplinary collaborative model.

 The development of HC and the integration of palliative medicine into health systems are among the most impactful parts of medical reform.^11^ Since the New Medical Reform of China was published in 2009, the government has accelerated the construction of primary healthcare institutions and promoted the development of modern HC. The Chinese National Commission conducted modern experimental work in HC in 5 cities across China in 2017^12^ and expanded this work to 87 cities in 2019^13^ (in step with the principles of the Healthy China 2030 strategic plan). Shanghai is a pioneer of the HC movement in China; today, it is home to an early-stage HC service system with localized characteristics.^14^

 Healthcare provider awareness and services are often regarded as significant facilitators or barriers in the development of HC. Physicians and nurses play an important role in HC teams, which include multidisciplinary professionals, such as social workers, psychiatrists, psychologists, and volunteers. Healthcare providers are expected to deliver maximal benefits to their patients, and their knowledge, attitudes, and practices in hospice care (KAPHC) are of vital importance to the development of HC. While more attention has been paid to the development of HC of late, it remains crucial for healthcare providers to strengthen their knowledge of and attitudes toward HC.^15^ Along these lines, training in HC is currently in high demand by most healthcare providers; in particular, there is a demand to learn knowledge and skills related to living wills and other legal concerns, techniques for stress relief, and social work.^16^ The insufficient awareness about the processes of life and death and the lack of innovative teaching methods in China make the situation even worse.^17^ Existing studies on HC were mostly conducted with physicians (especially oncologists) or nurses and often advise that more attention should be paid to educating healthcare providers in HC and EOL care to increase their self-efficacy in these areas.^18,19^ Relatively few studies have assessed KAP in healthcare providers and effective strategies for barriers to developing HC in China.

 Accordingly, we conducted a novel large-scale empirical study to comprehensively evaluate healthcare provider KAPHC based on an indigenized KAPHC scale published by our research team.^20^ Then, we performed a linear regression and Pearson’s (*r*) correlation analysis to uncover related influencing factors and their correlations. Ultimately, we sought to discover the problems in delivering HC and develop corresponding solutions rooted in improving healthcare provider KAPHC with targeted training. In particular, this study focused on the KAP of healthcare providers as a prerequisite to improve HC quality. Notably, this study provides an evidence-based scale and data that can be used to conduct similar inquiries in other areas of China. This study has theoretical and practical implications for improving the quality of life of terminal patients and their families and establishing a scientific basis for the development of high-quality HC service.

## Methods

###  Questionnaires

 This study was designed as a cross-sectional study with an anonymous questionnaire survey. Participants were informed that participation was voluntary and were required to give informed consent. We adopted the indigenized healthcare provider KAPHC scale, which was scientifically constructed by Shu et al^20^ and which proved to have good reliability and validity. The scale comprised four parts: demographic characteristics (11 items), knowledge (15 items), attitudes (24 items with 5 sub-concepts), and practices (2 sub-concepts, confidence of practices and self-reported practices, which each comprised 11 items) in HC. Only healthcare providers with HC experience were asked to give responses to survey items on self-reported practices (see Supplementary file 1). Scores for each subcategory were calculated separately. Regarding the knowledge scale, each item was marked as “right” (1) or “wrong” (0), based on previous widely used scales and expert advice. Meanwhile, the attitudes section was measured using a five-point Likert scale, from totally agree (5) to totally disagree (1). The score of negative topics (Dimensions 1 and 4) was corrected in reverse before calculation. The other two sections—confidence and self-reported practices—were also measured using a five-point Likert scale, from rather confident (5) to rather non-confident (1) and always (5) to never (1).

###  Data Collection

 In order to establish a systematic and comprehensive understanding of the status of healthcare provider KAPHC in Shanghai, we designed a survey that covered all HC institutions between November 1 and December 31, 2019. For each investigated institution, 30 healthcare providers were chosen randomly, including physicians, nurses, administrators, and other health technicians, and the sample size was expanded by an extra 5% to avoid invalid data. The inclusion criteria of the study were as follows: healthcare providers who (1) were employed in the units at the time of the study; (2) were certified physicians, nurses, health technicians, and administrators; and (3) volunteered to participate in this investigation and gave informed consent. The exclusion criteria were as follows: healthcare providers who (1) were not employed or certified in the sampled units and (2) refused to participate or were unable to independently complete the survey. The anonymous, cross-sectional questionnaire survey was conducted through SO JUMP, a professional online questionnaire survey platform used by a large number of companies and individuals. To minimize the probable bias, strict quality control was conducted throughout the process. A concentrated training was held at the municipal level for all quality controllers before the investigation, and a back-to-back quality control inspection on the questionnaire was conducted by the research group.

###  Statistical Methods

 Statistical analyses were performed using the commercial software IBM SPSS Statistics 24.0 (IBM Corporation, Armonk, NY). Descriptive statistics were used to analyze the participants’ demographic characteristics and KAP scores. Continuity variables were described by mean ± standard deviation (SD) and categorical variables by frequency and proportion. A linear regression analysis was used to assess associated factors of healthcare provider KAP. A positive standardized regression coefficient (*β*) was used to indicate a protective factor and negative one to indicate a risk factor. A larger |*β*| suggested greater effects on the dependent variable (0<|*β*|<1). Pearson’s (*r*) analysis was used to explore correlations between every two dimensions among knowledge, attitudes, confidence, and self-reported practices. A larger |*r*| indicated a stronger correlation and an |*r*| closer to 0 indicated a weaker correlation (0<|*r*|<1). The test level was *α*= 0.05 and all *P* values represented bilateral probability.

## Results

###  General Information and Demographic Characteristics

 A total of 7074 questionnaires were distributed to 223 selected health institutions from across Shanghai’s districts. In total, 7027 valid questionnaires were completed, with an effective response rate of 99.34%. The results verified that the KAPHC scale had good reliability and validity and was applicable to the KAP of the large sample of healthcare providers we surveyed (notably, their KAP aligned with China’s stage of HC development). Regarding knowledge, the Cronbach’s *α* coefficient was 0.746, the Kaiser–Meyer–Olkin was 0.882, and Bartlett’s test of sphericity was 13 580.322 (*P* < .001). The Cronbach’s *α* coefficients of attitudes, confidence, and self-reported practices were 0.851, 0.937, and 0.953, respectively. The Kaiser–Meyer–Olkin of the three parts were 0.919, 0.952, and 0.955, respectively. Bartlett’s tests of sphericity were 78 752.809 (*P* < .001), 5218.516 (*P* < .001), and 28 379.781 (*P* < .001), respectively.

 Most participants were female (79.22%), 62.42% were between 30-50 years of age, 68.14% had a bachelor’s degree or above, 38.17% were nurses, and 34.75% were physicians. More than half (79.18%) had witnessed the process of death of an EOL patient. In terms of participation, 42.44% of healthcare providers had provided HC and 57.49% of them were willing to provide HC. The most popular reason respondents were willing to provide HC was a sense of responsibility (75.50%); meanwhile, the most popular reason respondents were not willing to provide HC was pressure (73.52%). Table 1 details the respondents demographic characteristics.

**Table 1:** Demographic Characteristics of Participants (N = 7027)

**Characteristic**	**No. (%)**
Gender	
Male	1460 (20.78)
Female	5567 (79.22)
Age	
≤30	2094 (29.80)
30-50	4386 (62.42)
>50	547 (7.78)
Educational status	
Bachelor or above	4788 (68.14)
High school or vocational college	1928 (27.44)
Junior middle school or less	311 (4.42)
Marriage status	
Unmarried	1341 (19.08)
Married	5511 (78.43)
Divorced or widowed	175 (2.49)
Nationality	
Han	6901 (98.21)
Minorities	126 (1.79)
Religious belief	
None	6172 (87.83)
Other	855 (12.17)
Work specialty	
Administrator	905 (12.88)
Physician	2442 (34.75)
Nurse	2682 (38.17)
Other	998 (14.20)
Profession title	
Senior	461 (6.56)
Intermediate	3295 (46.89)
Junior	2785 (39.63)
None	486 (6.92)
Experience of death witness	
Yes	5564 (79.18)
No	1463 (20.82)
Experience in providing HC	
Yes	2982 (42.44)
No	4045 (57.56)
Willingness of providing HC	
Yes	4040 (57.49)
No	2987 (42.51)
If yes, your main consideration is	
It’s task from superior	541 (13.39)
It’s my duty	3050 (75.50)
My religious belief	41 (1.01)
It’s charitable	408 (10.10)
If no, your main consideration is	
It’s stressful	2196 (73.52)
Low salary	415 (13.89)
Unvalued	59 (1.98)
Meaningless	66 (2.21)
Blind-alley job	251 (8.40)

Abbreviation: HC, hospice care.

###  Knowledge

 The mean knowledge score of respondents was 8.90 ± 2.632; response accuracy was 59.30%. Table 2 details the questions and scores. Question 14, “HC should be provided by a multi-professional HC team that includes general physicians,” had the highest rate of correct responses (93.88%), whereas Question 1, “The provision of HC requires emotional detachment,” turned out the lowest rate of correct responses (16.58%). The question with second lowest rate of correct responses (26.57%) occurred with Question 7, “During the terminal stages of an illness, respiratory depression medicine are appropriate for certain treatments of severe dyspnea.” In total, 8 items had a rate of correct responses below 60.00%.

**Table 2:** Healthcare Providers’ Knowledge of Hospice Care (N = 7027)

**Items**	**Correct Number (%)**
1. The provision of HC requires emotional detachment.	1165 (16.58)
2. Psychological, social, and spiritual problems are paramount to the HC team who give appropriate consultation and management.	6423 (91.40)
3. Three steps make up the WHO analgesic ladder.	6307 (89.75)
4. The HC team provides bereavement support for the family after the patient’s death.	5184 (73.77)
5. Home HC is in line with China’s folk customs.	4673 (66.50)
6. For children’s bereavement care, children can attend funerals and even participate in preparations.	2615 (37.21)
7. During the terminal stages of an illness, respiratory depression medicine are appropriate for certain treatment of severe dyspnea.	1867 (26.57)
8. To use Mirabilite in Shenque acupoint application can relieve ascites.	4034 (57.41)
9. Pain threshold is lowered by fatigue or anxiety.	2906 (41.34)
10. Men generally reconcile their grief more quickly than women.	2205 (31.38)
11. Individuals who are taking opioids should also follow a bowel regime.	4156 (59.14)
12. To strengthen the construction of HC institutions was written into the “Healthy China 2030” strategic plan.	5800 (82.54)
13. Morphine point injections can be used to relieve cancer pain in the terminal period.	4965 (70.66)
14. The most authoritative guidelines on health care planning recommend that HC should be provided by: (1) multi-professional HC team that includes the family’s general physicians, (2) general physicians, (3) multi-professional hospital team led by a pain therapist, (4) specialized nursing staff in collaboration with an anesthetist, and (5) specialized nursing staff.	6597 (93.88)
15. The purposes of melodic therapy are not including (1) to relieve physical pain, (2) entertainment, (3) to express emotions, (4) to evoke memories, and (5) to comfort grief.	3611 (51.39)
**Total**	62 508 (59.30)

Abbreviations: HC, hospice care; WHO, World Health Organization.

 The linear regression analysis (Table 3) evidences that the factors associated with higher knowledge scores were higher education level (*β*= 0.122, *P* < .001), higher professional title (*β*= 0.039, *P*= .012), experience in witnessing death (*β*= 0.044, *P*= .001), experience in providing HC (*β*= 0.064, *P* < .001), and willingness to provide HC (*β*= 0.165, *P* < .001). Nurses scored lower on the knowledge scale (*β*= -0.077, *P* < .001) than others. In the regression model, Tolerance >0.1 and 0 < variance inflation factor (VIF) < 10, indicating that no significant collinearity existed.

**Table 3:** Linear Regression Analysis of Knowledge, Attitudes, and Practices in Hospice Care

**Model**	**Variables**	**B**	**SE**	**β**	**t**	***P***	**Tolerance**	**VIF**
Knowledge	(Constant)	6.765	0.273	-	24.806	<.001**	-	-
	Educational status	0.566	0.060	0.122	9.433	<.001**	0.794	1.260
	Work specialty							
	Group 1 (Administrator)	Reference						
	Group 2 (Physician)	0.029	0.101	0.005	0.286	.775	0.398	2.516
	Group 3 (Nurse)	-0.419	0.107	-0.077	-3.906	<.001**	0.337	2.968
	Group 4 (Other)	0.048	0.123	0.006	0.393	.694	0.496	2.015
	Profession title	0.141	0.056	0.039	2.515	.012*	0.559	1.789
	Experience of death witness							
	No	Reference						
	Yes	0.288	0.085	0.044	3.387	.001**	0.767	1.304
	Experience in providing HC							
	No	Reference						
	Yes	0.343	0.066	0.064	5.193	<.001**	0.857	1.167
	Willingness of providing HC							
	No	Reference						
	Yes	0.877	0.064	0.165	13.694	<.001**	0.912	1.097
Attitudes	(Constant)	81.031	1.122	-	72.224	<.001**	-	-
	Educational status	0.830	0.247	0.041	3.359	.001**	0.794	1.260
	Work specialty							
	Group 1 (Administrator)	Reference						
	Group 2 (Physician)	-1.073	0.414	-0.045	-2.590	.010*	0.398	2.516
	Group 3 (Nurse)	-0.403	0.441	-0.017	-0.913	.361	0.337	2.968
	Group 4 (Other)	-0.272	0.506	-0.008	-0.537	.591	0.496	2.015
	Experience of death witness							
	No	Reference						
	Yes	1.496	0.350	0.054	4.274	<.001**	0.767	1.304
	Experience in providing HC							
	No	Reference						
	Yes	0.997	0.272	0.044	3.665	<.001**	0.857	1.167
	Willingness of providing HC							
	No	Reference						
	Yes	8.297	0.264	0.362	31.479	<.001**	0.912	1.097
Confidence	(Constant)	36.412	0.777	-	46.892	<.001**	-	-
	Gender							
	Female	Reference						
	Male	0.815	0.232	0.042	3.516	<.001**	0.839	1.192
	Age	0.564	0.204	0.041	2.759	.006**	0.543	1.843
	Work specialty							
	Group 1 (Administrator)	Reference						
	Group 2 (Physician)	-0.695	0.287	-0.042	-2.422	.015*	0.398	2.516
	Group 3 (Nurse)	0.005	0.305	0.000	0.017	.986	0.337	2.968
	Group 4 (Other)	-0.039	0.350	-0.002	-0.111	.912	0.496	2.015
	Experience of death witness							
	No	Reference						
	Yes	0.755	0.242	0.039	3.116	.002**	0.767	1.304
	Experience in providing HC							
	No	Reference						
	Yes	1.325	0.188	0.084	7.040	<.001**	0.857	1.167
	Willingness of providing HC							
	No	Reference						
	Yes	5.393	0.182	0.341	29.560	<.001**	0.912	1.097
Self-reported practices	(Constant)	36.593	1.640	-	22.317	<.001**	-	-
	Gender							
	Female	Reference						
	Male	0.764	0.387	0.038	1.972	.049*	0.813	1.230
	Educational status	0.653	0.294	0.044	2.225	.026*	0.795	1.258
	Marriage status							
	Group 1 (Unmarried)	Reference						
	Group 2 (Married)	0.969	0.443	0.047	2.186	.029*	0.646	1.547
	Group 3 (Divorced or widowed)	2.672	0.981	0.053	2.723	.007**	0.813	1.230
	Work specialty							
	Group 1 (Administrator)	Reference						
	Group 2 (Physician)	1.964	0.653	0.117	3.008	.003**	0.394	2.539
	Group 3 (Nurse)	0.757	0.507	0.046	1.494	.135	0.323	3.091
	Group 4 (Other)	-0.154	0.722	-0.005	-0.213	.831	0.633	1.579
	Experience of death witness							
	No	Reference						
	Yes	2.896	0.574	0.094	5.043	<.001**	0.870	1.149
	Willingness of providing HC							
	No	Reference						
	Yes	4.953	0.321	0.275	15.409	<.001**	0.956	1.046

Abbreviations: HC, hospice care; SE, standard error; VIF, variance inflation factor. *P < .05,**P < .01.

###  Attitudes

 The mean score of the attitudes scale was 89.04 ± 11.320 and the total scoring rate was 74.20%. Table 4 presents the attitudes scale items and their mean scores. Among the different dimensions, “Perception of the benefits for the life quality promotion” had the highest mean score (4.02 ± 0.635), whereas “Perception of the threats from the worsening conditions of advanced patients” had the lowest score (3.04 ± 0.841). Question 8, “Having care and accompany by medical team enhance quality of life” had the highest mean score (4.42 ± 0.728); by contrast, Question 19, “Advanced patients’ complex symptoms are barriers to providing HC” had the lowest mean score (2.51 ± 1.068).

**Table 4:** Healthcare Providers’ Attitudes Toward Hospice Care (N = 7027)

**Items**	**Mean ± SD**
Perception of the threats from the worsening conditions of advanced patients is:	3.04 ± 0.841
1. Uncomfortable to take care of advanced cancer patients.	3.51 ± 1.207
2. Hopeless for the cure.	2.70 ± 1.173
3. Unable to easily face dying process and distress.	3.11 ± 1.160
4. Makes me feel weak.	3.01 ± 1.221
5. I feel guilty when amine patient dies.	2.89 ± 1.182
Perception of the benefits for the life quality promotion is:	4.35 ± 0.671
6. Able to promote life quality and keep the dignity.	4.38 ± 0.814
7. Able to die peacefully and have a good death.	4.39 ± 0.769
8. Having care and accompany by medical team.	4.42 ± 0.728
9. Emotional support.	4.35 ± 0.755
10. Able to have family support.	4.24 ± 0.804
Perception of the benefits for better death preparation is:	4.02 ± 0.635
11. Respect for patient’s religion and burial rites.	4.34 ± 0.788
12. Help to die at home.	3.64 ± 0.968
13. Better communication with advanced patients.	4.19 ± 0.787
14. Help medical staff to take care of patients better.	4.27 ± 0.739
15. Avoid the idea of euthanasia.	3.68 ± 0.952
Perception of the barriers to provide HC is:	3.31 ± 0.806
16. Shorten patient’s life, just like euthanasia.	3.74 ± 1.149
17. No active treatment for physical symptoms.	3.39 ± 1.166
18. Make patients feel hopeless.	3.77 ± 1.123
19. Advanced patients have many complex symptoms.	2.51 ± 1.068
20. Keep providing long-term HC service will lose enthusiasm.	3.14 ± 1.159
Subjective norms for provision of HC:	3.85 ± 0.733
21. It is meaningful.	4.26 ± 0.793
22. I experienced the death of my family member, which affected me to provide HC.	3.50 ± 1.042
23. It is a part of duty on medical staff.	3.96 ± 0.902
24. With the approval and support of department leader, colleagues, relatives and friends, I was encouraged to provide HC.	3.67 ± 0.966
**Total**	89.04 ± 11.320

 The linear regression analysis (Table 3) evidences that the following variables significantly correlated with higher attitudes scores: higher education level (*β*= 0.041, *P*= .001), experience in witnessing death (*β*= 0.054, *P* < .001), experience in providing HC (*β*= 0.044, *P* < .001), and willingness to provide HC (*β*= 0.362, *P* < .001). Physicians had lower attitudes scores (*β*= -0.045, *P*= .010) than others.

###  Confidence and Self-reported Practices

 Among all participants, there were 2982 (42.44%) healthcare providers with self-reported HC experience; these providers completed the self-reported practices scale. The total scores for confidence and self-reported practices toward HC were 40.68 ± 7.822 and 41.55 ± 8.145, respectively; the total scoring rates were 73.96% for confidence and 75.55% for practices. Table 5 presents the mean scores of the confidence and self-reported practices scale items. Question 8, “Create good relationship between the medical staff and family members,” had the highest mean score (3.87±0.849, 3.99 ± 0.797), whereas Question 11, “Guide the management of afterwards and funeral preparation for families,” had the lowest (3.55 ± 0.996, 3.43 ± 1.048). The data analysis of Table 6 shows that correlations existed (*P* < .05) among two of the four dimensions (ie, knowledge, attitudes, confidence, and self-reported practices); more specifically, HC practices were strongly correlated with confidence (*r* = 0.648, *P* < .001) and moderately correlated with attitudes (*r *= 0.463, *P* < .001).

**Table 5:** Scores of Healthcare Providers’ Confidence and Self-reported Practices in Hospice Care

**Items**	**Confidence** **Mean ± SD** **(N = 7027)**	**Self-reported Practices** **Mean ± SD** **(N = 2982)**
1. Alleviate pain and discomfort of dying patients.	3.60 ± 0.939	3.96 ± 0.823
2. Make pain assessment of patients.	3.84 ± 0.849	3.89 ± 0.905
3. Reduce unnecessary treatment costs.	3.68 ± 0.880	3.77 ± 0.909
4. Satisfy the physical and mental needs of dying patients.	3.62 ± 0.951	3.88 ± 0.835
5. Explain the expected dying process to the patient’s family.	3.63 ± 0.925	3.67 ± 0.930
6. Tell family specific things they can do to provide meaningful service to patients.	3.85 ± 0.825	3.82 ± 0.845
7. Understand the wishes and pain of family to help them.	3.83 ± 0.859	3.84 ± 0.830
8. Create good relationship between the medical staff and family members.	3.87 ± 0.849	3.99 ± 0.797
9. Coordinate the media resources of medical, social, psychological and spiritual care.	3.64 ± 0.939	3.62 ± 0.992
10. Help risk grieving families to get through better.	3.58 ± 0.954	3.68 ± 0.914
11. Guide the management of afterwards and funeral preparation for families.	3.55 ± 0.996	3.43 ± 1.048
**Total**	40.68 ± 7.822	41.55 ± 8.145
**Average**	3.70 ± 0.711	3.78 ± 0.740

Abbreviation: SD, standard deviation.

**Table 6:** Pearson’s Correlation Analysis of Knowledge, Attitudes, and Practices in Hospice Care

**Variables**	**Knowledge**	**Attitudes**	**Confidence**	**Self-reported Practices**
Knowledge	1	0.309**	0.258**	0.235**
Attitudes	0.309**	1	0.590**	0.463**
Confidence	0.258**	0.590**	1	0.648**
Self-reported practices	0.235**	0.463**	0.648**	1

*P < .05,**P < .01.

 Being male (*β *= 0.042, *P* < .001, *β*= 0.038, *P*= .049), having experience in witnessing death (*β*= 0.039, *P*= .002, *β*= 0.094, *P* < .001), and willingness to provide HC (*β*= 0.341, *P* < .001, *β*= 0.275, *P* < .001) were associated with higher overall scores in confidence and self-reported practices. Physicians scored lower on the confidence scale (*β *= -0.042, *P*= .015) and higher on the self-reported practices scale (*β *= 0.117, *P* = .003) (Table 3).

## Discussion

###  Knowledge

 The mean knowledge score of healthcare providers regarding HC was 8.90 out of 15 (59.30%), indicating that the healthcare providers had insufficient knowledge of HC; this is generally consistent with other domestic studies^21,22^_._ This lack of knowledge may mainly be due to deficits in HC education. Physicians from different countries expressed that current undergraduate and postgraduate programs did not provide them with sufficient education on HC.^23^ Along these same lines, Knapp et al^24^ showed that 279 American pediatric nurses scored 10.9 out of 20 (54.50%) on the Palliative Care Quiz for Nursing, and Abudari et al^25^ reported that 395 nurses from 19 cities in Saudi Arabia scored 9.06 out of 20 (45.30%) on the Palliative Care Quiz for Nursing.

 Our study found that healthcare providers had a basic level of knowledge about HC, its policies, and its service goals, but that they were generally unaware of best practices for psychological and spiritual care. To be sure, psychological and spiritual care is very important: sufficient evidence exists to suggest that healthcare providers can be extremely helpful by providing support, advice, and encouragement to patients by listening and talking to them. Notably, a handful of our respondents believed that HC required emotional detachments; it should be emphasized that such psychological and spiritual care is ineffective if healthcare providers are emotionally detached^26^—education in HC should work to clear up this misconception. The lack of awareness regarding spiritual care is not surprising—there is a lack of training on spiritual care in China generally.^27^ Furthermore, the lower therapeutic accuracy of pain thresholds for some diseases reflected healthcare providers’ lack of knowledge about pain and other symptoms management. Additionally, the importance of children’s participation and the application of traditional Chinese medicine (TCM) in HC were also poorly recognized. Treatments that combine TCM and Western medicine can reduce the suffering of EOL patients, especially those with cancer.^28^

 Studies have shown that healthcare providers demonstrated better knowledge and attitudes in HC after being trained in the field.^29,30^ Therefore, healthcare providers in different positions should complete professional HC and palliative care training to fill knowledge gaps, especially regarding psychological and spiritual care, the management of pain and other symptoms, and the application of TCM.

###  Attitudes

 Our study revealed that healthcare providers had moderate attitudes toward HC; the score in our study was 89.04 out of 120 (74.20%), which is higher than the scores in other domestic studies.^31,32^ Regarding international studies that used the Frommelt Attitude Toward Care of the Dying Scale, Lange et al^33^ reported a score of 129 out of 150 (86.00%) for 355 oncology nurses in the United States, Abudari et al^25^ reported a score of 111.66 out of 150 (74.44%) in Saudi Arabia, and another study reported a score of 71.67% for 190 nurses in Japan.^34^ These differences are likely attributable to international differences in cultural approaches to the process of life and death and HC training.^34^

 Our study found that healthcare providers highly agreed that HC had many benefits and especially affirmed its ability to enable patients to access people-oriented care provided by multidisciplinary teams that allowed them to die with a relatively good quality of life and with dignity. However, healthcare providers only partly agreed with the idea that HC can help people die at home and avoid considering euthanasia; this negative attitude may impede the development of home-based HC to a certain degree. In addition, healthcare providers reported that the main barrier to providing HC was that advanced cancer patients had many complex symptoms. This echoes their lack of relevant knowledge and is consistent with studies by Liu et al,^35^ Sun et al,^36^ and Kuang et al.^37^ Healthcare providers reported that they often felt sad and experienced a sense of loss when providing HC; sometimes they said they even felt guilty when patients died. Traditional medical education mainly focuses on the treatment of diseases rather than comforting or relieving the patient suffering; this may cause physicians to regard incurable diseases as “medical failures.”^35^ The related negative attitudes of healthcare providers toward complex symptoms highlights a trend of poor medical skills in symptom control and management. A lack of professional training in HC will inevitably affect provider ability and attitude, which can cause psychological pressure and distress—key reasons why healthcare providers in our study were unwilling to participate in HC. Promoting professional training in HC for healthcare providers can improve their attitudes toward HC^38,39^ (and thus help them deal calmly with death^40^) and reduce or alleviate the barriers to providing HC services. Designing and offering professional training in HC will remain a worthwhile endeavor.

###  Confidence and Self-reported Practices

 The mean score of the confidence scale was 40.68 out of 55 (73.96%), which indicated that healthcare provider confidence levels leaned toward “confident” (3.70 ± 0.711). Regarding the self-reported practices of the providers with HC experience, the mean score was 41.55 out of 55 (75.55%) and the frequency leaned toward “usually” (3.78 ± 0.740). The results showed that most healthcare providers needed support in providing HC; this is consistent with some previous studies, such as a cross-sectional descriptive study on nurses in Mongolia^19^ and another physician-related study in Vietnam.^29^ Domestically, our result was higher than that of Gai et al,^41^ who reported that 128 oncology nurses in Shenzhen scored 64.81 out of 100 (64.18%). However, our result was lower than that of Wang et al,^21^ who reported that 1037 oncology physicians and nurses in Shandong scored 3.92 out of 5 (78.40%). This was mainly due to different questionnaire design models and participants. We also found that the frequency of self-reported practices was consistent and positively correlated with the outcomes of the confidence scale (*r*= 0.648, *P*< .001). Healthcare providers had better confidence and practices in maintaining stronger doctor–patient relationships, understanding and responding to family members’ wishes and concerns, assessing patients’ pain, and satisfying patients’ physical and mental needs. Participants usually alleviated the pain and discomfort of dying patients, but they were not confident about their abilities, mainly because they lacked professional skills in pain control. Moreover, healthcare providers had low confidence about guiding death and funeral preparations and their related scores for practices were also low. The traditional doctrines of Confucianism, Taoism, and Buddhism have deeply impacted the Chinese view of life and death and continue to affect contemporary life,^42^ such as the taboos of discussing death and inappropriate view of life and death. Without a professional education in death, some healthcare providers feel helpless in regard to discussing patient deaths with family members.^43^ Comprehensive education on the process of life and death should be given not only to patients and their families but also their caregivers. Healthcare providers who had a scientific outlook on death were able to help EOL patients and their families face death more positively and with dignity.^44^ The experiences of developed countries like the United Kingdom have proved that life-and-death-related research can make HC more acceptable to healthcare providers, patients, and family members. Additionally, education on death has brought great improvements to the development of HC.^45^

###  Influencing Factors of KAPHC

 There were positive correlations among the dimensions of KAP. Healthcare providers with higher scores in knowledge and attitudes usually expressed more confidence and practiced more frequently, which indicated a strong correlation between practices and confidence. The linear regression analysis suggested that healthcare providers with experience in witnessing death or a high willingness to deliver HC scored significantly higher on the KAPHC scale; this illustrates that work experience and good professional identity promote HC practices. While most healthcare providers fully recognize the need to promote quality of life, 42.51% of respondents were unwilling to participate in HC delivery because of the high level of pressure it involved (reason cited by 73.52% of respondents unwilling to deliver HC). Thus, relieving work stress is an urgent issue that should be solved to encourage healthcare providers to deliver HC. In addition, the government and HC institutions should introduce and implement innovative payment and incentive policies to encourage providers to work actively and enthusiastically in HC.

 The study revealed that the KAPHC scores of healthcare providers were significantly influenced by their work specialties. Physicians demonstrated better practices and knowledge but worse attitudes. In comparison, nurses reported better attitudes but poorer practices and professional knowledge. Additionally, gender, age, marital status, education level, and professional title were also influencing factors, as other studies have evidenced.^46,47^ Healthcare providers at all levels need professional HC training tailored to their characteristics, which can improve their own perceptions of their levels of KAPHC as well as their real relative levels of KAPHC.

###  Limitations 

 This study had several limitations. Firstly, Shanghai is a developed region that is pioneering HC in China; accordingly, the KAP levels of the healthcare providers in Shanghai may be better than those of providers in other areas of China. The results should be verified by work conducted across different areas of China. Secondly, some bias may appear due to the format of the question design: the questions for the attitudes scale were mainly subjective items and the confidence and practices items were self-reported. Lastly, the study did not specify how the influencing factors affected the KAPHC scores of the healthcare providers; further research is needed to explore this in depth.

## Conclusion

 This study comprehensively investigated healthcare providers’ knowledge, attitudes, confidence regarding practices, and self-reported practices in HC and the correlations between these different dimensions. We found that the KAPHC scale was applicable to the KAP of the large sample of healthcare providers we surveyed, which spoke to China’s current state of HC. This study accordingly offers insights on how to conduct similar measurements in other regions of China. Healthcare providers demonstrated insufficient knowledge in HC, especially related to psychological care, the management of pain and other symptoms, and the application of TCM. Meanwhile, the outcomes of attitudes, confidence, and self-reported practices were moderate. The KAPHC of healthcare providers varied significantly with gender, education level, professional title, work specialty, HC experience, and willingness to provide HC. Additionally, there were close correlations among KAP dimensions. Ultimately, the study evidences that the government and HC organizations would do well to scale up targeted HC training for healthcare providers with different characteristics to improve their KAPHC; moreover, they should also introduce innovative payment and incentive policies to encourage providers to work enthusiastically in HC. Universal access to high-quality HC can only be achieved through the participation of well-trained, supported, and motivated healthcare providers.

## Acknowledgements

 Firstly, we gratefully acknowledge the participation of all healthcare institutions and providers. Secondly, we appreciate the support of Shanghai Municipal Health Commission and grants from the Nature Science Foundation of Shanghai [22ZR1461400], Humanities and Social Science Research Planning Fund of the Ministry of Education [20YJAZH045], Philosophy and Social Science Planning Project of Shanghai [2019BGL032], and Pujiang Talent Program of Shanghai [2019PJC099]. Additionally, we would like to thank Editage (https://www.editage.cn/) for English language editing.

## Ethical issues

 This study was approved by the ethics committees of Shanghai Ninth People’s Hospital (ref: SH9H-2021-T11-1).

## Competing interests

 Authors declare that they have no competing interests.

## Authors’ contributions

 LJ secured the funding to conduct this work and was responsible for conceptualizing the study. XT and YX collected and analyzed the data, ZS contributed to the design of the scale, XT drafted the initial manuscript, and MT revised and polished the manuscript. All authors were responsible for critical revisions to and approval of the final manuscript.

## Funding

 This work was supported by grants from Nature Science Foundation of Shanghai [22ZR1461400], Humanities and Social Science Research Planning Fund of the Ministry of Education [20YJAZH045], Philosophy and Social Science Planning Project of Shanghai [2019BGL032], and Pujiang Talent Program of Shanghai [2019PJC099].

## Supplementary files

Supplementary file 1. Health Providers’ Knowledge, Attitude and Practice of Hospice Care (KAPHC) Scale in China.
