# Supplementary material for: Healthcare Provider Knowledge, Attitudes, and Practices in Hospice Care and Their Influencing Factors: A Cross-sectional Study in Shanghai
Source: Int J Health Policy Manag. 2022 Aug 15;11(12):3090–100. doi: 10.34172/ijhpm.2022.6525 (PMC10105208; doi:10.34172/ijhpm.2022.6525)
Supplement: Supplementary file 1 — Health Providers’ Knowledge, Attitude and Practice of Hospice Care (KAPHC) Scale in China. [file ijhpm-11-3090-s001.pdf]

Article title: Healthcare Provider Knowledge, Attitudes, and Practices in Hospice Care and Their Influencing Factors: A Cross-sectional Study in Shanghai

Journal name: International Journal of Health Policy and Management (IJHPM)

Authors' information: Xiaohan Teng[1,2]¶, Maocheng Tang[3]¶, Limei Jing[1]*, Yifan Xu1, Zhiqun Shu[4]

[1]School of Public Health, Shanghai University of Traditional Chinese Medicine,

Shanghai, China.

[2]Longhua Hospital, Shanghai University of Traditional Chinese Medicine, Shanghai, China.

[3]Department of Traditional Chinese Medicine, Shandong College of Traditional Chinese Medicine, Yantai, China.

[4]Shanghai Ninth People's Hospital, Shanghai Jiaotong University School of Medicine, Shanghai, China.

¶ Both authors contributed equally to this paper.

(Corresponding author: limei_jing@163.com)

Supplementary file 1. Health Providers' Knowledge, Attitude and Practice of Hospice Care (KAPHC) Scale in China

Demographic Characteristics

1. Gender: ○ Male ○ Female

2. Age: ________(years old)

3. Marriage status: ○ Unmarried ○ Married ○ Divorced or widowed

4. Nationality: ○ Han ○ Minorities

5. Religious Belief: ○ Buddhism ○ Taoism ○ Christian ○ Catholicism ○ Mohammedanism ○ None

6. Work Specialty: ○ Administrator ○ Physician ○ Nurse ○ Other

7. Educational status: ○ Bachelor or above ○ High school or vocational college ○ Junior middle school or less

8. Profession title: ○ Senior ○ Intermediate ○ Junior ○ None

9. Have you ever seen the death process of end-of-life patient or relatives? ○ Yes ○ No

10. Have you provided any hospice care service? ○ Yes ○ No

11. Are you willing to participate in hospice care? ○ Yes ○ No

11.1 If yes, your main consideration is:

○ It's task from superior. ○ It's my duty. ○ My religious belief. ○ It's charitable.

11.2 If no, your main consideration is:

○ It's stressful. ○ Low salary. ○ Unvalued. ○ Meaningless. ○ Blind - alley job.

Knowledge

Please indicate the right answer and fill in the corresponding number.

Items	Option
1. The provision of HC requires emotional detachment. 1) Yes 2) No 3) Don't know	
2. Psychological, social, and spiritual problems are paramount to the HC team who give appropriate consultation and management. 1) Yes 2) No 3) Don't know	
3. Three steps make up the WHO analgesic ladder. 1) Yes 2) No 3) Don't know	
4. The HC team provides bereavement support for the family after the patient's death. 1) Yes 2) No 3) Don't know	
5. Home HC is in line with China's folk customs. 1) Yes 2) No 3) Don't know	
6. For children's bereavement care, children can attend funerals and even participate in preparations. 1) Yes 2) No 3) Don't know	
7. During the terminal stages of an illness, respiratory depression medicine are appropriate for certain treatment of severe dyspnea. 1) Yes 2) No 3) Don't know	
8. To use Mirabilite in Shenque acupoint application can relieve ascites. 1) Yes 2) No 3) Don't know	
9. Pain threshold is lowered by fatigue or anxiety. 1) Yes 2) No 3) Don't know	
10. Men generally reconcile their grief more quickly than women. 1) Yes 2) No 3) Don't know	
11. Individuals who are taking opioids should also follow a bowel regime. 1) Yes 2) No 3) Don't know	
12. To strengthen the construction of HC institutions was written into the "Healthy China 2030" strategic plan. 1) Yes 2) No 3) Don't know	
13. Morphine point injections can be used to relieve cancer pain in the terminal period. 1) Yes 2) No 3) Don't know	
14. The most authoritative guidelines on health care planning recommend that HC should be provided by: 1) multi-professional HC team that includes the family's general physicians 2) general physicians 3) multi-professional hospital team led by a pain therapist 4) specialized nursing staff in collaboration with an anesthetist 5) specialized nursing staff	
15. The purposes of melodic therapy are not including: 1) to relieve physical pain 2) entertainment 3) to express emotions 4) to evoke memories 5) to comfort grief	

Attitudes

Which answer corresponds to your own personal feelings about the attitude presented, please fill in the corresponding number in the blank. The meaning of number is:

1 = totally disagree, 2 = partly disagree, 3 = neutral/nonsense, 4 = partly agree, 5 = totally agree.

**Items**	**1**	**2**	**3**	**4**	**5**
**Perception of the threats from the worsening conditions of advanced patients is:**					
1. Uncomfortable to take care of advanced cancer patients.					
2. Hopeless for the cure.					
3. Unable to easily face dying process and distress.					
4. Makes me feel weak.					
5. I feel guilty when amine patient dies.					
**Perception of the benefits for the life quality promotion is:**					
6. Able to promote life quality and keep the dignity.					
7. Able to die peacefully and have a good death.					
8. Having care and accompany by medical team.					
9. Emotional support.					
10. Able to have family support.					
**Perception of the benefits for better death preparation is:**					
11. Respect for patient's religion and burial rites.					
12. Help to die at home.					
13. Better communication with advanced patients.					
14. Help medical staff to take care of patients better.					
15. Avoid the idea of euthanasia.					
**Perception of the barriers to provide HC is:**					
16. Shorten patient's life, just like euthanasia.					
17. No active treatment for physical symptoms.					
18. Make patients feel hopeless.					
19. Advanced patients have many complex symptoms.					
20. Keep providing long-term HC service will lose enthusiasm.					
**Subjective norms for provision of HC:**					
21. It is meaningful.					
22. I experienced the death of my family member, which affected me to provide HC.					
23. It is a part of duty on medical staff.					
24. With the approval and support of department leader, colleagues, relatives and friends, I was encouraged to provide HC.					

Confidence and Self-report Practices

Please fill in the corresponding number in the blank, which corresponds to

1) your personal feelings about the confidence to provide hospice care services presented.

In confidence items the meaning of number is:

1 = rather non confident, 2 = non confident, 3 = neutral/nonsense, 4 = confident, 5 = rather confident.

2) And in self-report practices items, please fill in the correspondent to your actual work (how often you performed) if you have had the hospice working experience (**if don't have any experience, please skip this part**).

The meaning of number is:

1 = never, 2 = rarely, 3 = sometimes, 4 = usually, 5 = always.

**How much confidence do you have?**	**1**	**2**	**3**	**4**	**5**
1. Alleviate pain and discomfort of dying patients.					
2. Make pain assessment of patients.					
3. Reduce unnecessary treatment costs.					
4. Satisfy the physical and mental needs of dying patients.					
5. Explain the expected dying process to the patient's family.					
6. Tell family specific things they can do to provide meaningful service to patients.					
7. Understand the wishes and pain of family to help them.					
8. Create good relationship between the medical staff and family members.					
9. Coordinate the media resources of medical, social, psychological and spiritual care.					
10. Help risk grieving families to get through better.					
11. Guide the management of afterwards and funeral preparation for families.					
**If you have hospice working experience, how often you performed?**					
1. Alleviate pain and discomfort of dying patients.					
2. Make pain assessment of patients.					
3. Reduce unnecessary treatment costs.					
4. Satisfy the physical and mental needs of dying patients.					
5. Explain the expected dying process to the patient's family.					
6. Tell family specific things they can do to provide meaningful service to patients.					
7. Understand the wishes and pain of family to help them.					
8. Create good relationship between the medical staff and family members.					
9. Coordinate the media resources of medical, social, psychological and spiritual care.					
10. Help risk grieving families to get through better.					
11. Guide the management of afterwards and funeral preparation for families.					
